# Development of psychosis following sexual abuse:rape of an adolescent: A case study

**DOI:** 10.1192/j.eurpsy.2021.2120

**Published:** 2021-08-13

**Authors:** S. Sellami, H. Mami, R. Bouzid

**Affiliations:** Department Of Psychiatry, University hospital of Nabeul, Nabeul, Tunisia

**Keywords:** sodomy, psychosis, adolescent, rape

## Abstract

**Introduction:**

Several studies have mentioned the link between psychotrauma and psychosis. A direct causal link remains to be discussed.

**Objectives:**

Evaluate the link between sexual abuse and psychosis.

**Methods:**

We report the case of a male patient who developed schizophrenia following sodomy rape. We performed a literature review based on a PubMed search with the following keywords: “rape sodomy psychosis”.

**Results:**

Mr. M., 26 years old, with a personal psychiatric history of chronic psychosis evolving for 10 years, consulted us for follow-up of his schizophrenia. When he was 16, the patient was raped by sodomy by a 40-year-old man under stabbing threat. After this incident, the patient did not verbalize this trauma, he isolated himself, became irritable and aggressive and has had olfactory hallucinations. The symptomatology worsened until the age of 24 when the patient presented a delusional syndrome with a theme of persecution, mysticism, bewitchment by a mechanism of interpretation and visual hallucinations. Then,he was hospitalized in psychiatry for psychomotor instability, verbal hetero-aggression. He had been diagnosed with schizophrenia evolving over 9 years. Treatment with an antipsychotic: risperidone and valproic acid was started. The evolution was quickly favorable but the patient currently presents blunted affect, a sexual disinterest and a strong desire for revenge from his rapist. Treatment adjustment and psychotherapy would be considered.

**Conclusions:**

The onset of subsequent rape psychosis and the persistence of symptoms related to the trauma are arguments in favor of a direct causal link between sexual abuse and schizophrenia.

**Disclosure:**

No significant relationships.

